# Neural correlates of categorization uncertainty: evidence from a category induction task

**DOI:** 10.3389/fpsyg.2026.1556213

**Published:** 2026-03-25

**Authors:** Xueli Cai, Miao Zhang, Zhencai Chen, Mengxue Wu, Hong Tang, Jun Lei, Ming Lei, Yanhui Mao, Yifeng Wang

**Affiliations:** 1 Psychological Research and Counseling Center, Southwest Jiaotong University, Chengdu, Sichuan, China; 2 College of Humanities, Jiangxi University of Chinese Medicine, Nanchang, China; 3 Institute of Brain and Psychological Sciences, Sichuan Normal University, Chengdu, Sichuan, China

**Keywords:** categorization uncertainty, cingulate gyrus, fronto-parietal network, functional connectivity, similarity-based categorization

## Abstract

**Introduction:**

Categorization uncertainty occurs when novel stimuli are classified as category members or non-members, with frontal–parietal and insular regions involved in its monitoring, but graded uncertainty in similarity-based category induction—especially implicit processing without explicit confidence judgments—has not been well characterized.

**Methods:**

The present study investigated these mechanisms using a similarity-based category induction task combined with functional magnetic resonance imaging (fMRI). Participants simultaneously viewed three stimuli: two reference stimuli (S1 and S2) that defined a target category, and a probe stimulus (S3) for which they judged category membership. Levels of categorization uncertainty were manipulated by varying the degree of feature similarity between the probe and reference stimuli.

**Results:**

The fMRI data revealed that activity in the left fronto-parietal network—including the medial frontal gyrus (BA11), middle frontal gyrus (BA46), cingulate gyrus (BA32), inferior parietal lobule (BA40), and left insula (BA13)—increased systematically with higher categorization uncertainty. Notably, positive activations were observed in the left cingulate gyrus, middle frontal gyrus, and inferior parietal lobule, whereas negative activations were detected in the left medial frontal gyrus and insula. Psychophysiological interaction (PPI) analyses further demonstrated enhanced functional coupling between the left cingulate cortex and inferior parietal lobule under high uncertainty conditions.

**Discussion:**

These findings suggest that distributed fronto-parietal and insular systems support the processing of graded uncertainty during similarity-based category induction, even in the absence of explicit confidence judgments.

## 1 Introduction

Our categorization decisions and choices, for instance, distinguishing food toxicity, inherently involve varying levels of uncertainty and perceived risk (Grinband et al., 2006; Seger et al., 2015). These uncertainties arise when a decision requires incomplete information or when the probability or predictability of the outcome remains elusive (Murphy and Ross, 2010; Chen et al., 2014, 2016). Furthermore, uncertainty can arise psychologically when probabilities are estimable, but the categorization of a given stimulus remains ambiguous (Huettel et al., 2005; Daniel and Pollmann, 2010).

Categorization is indeed a pivotal and ever-evolving aspect of the decision-making process (Grinband et al., 2006; Chen et al., 2014). The neural mechanisms underlying categorization under uncertainty have been investigated in previous studies (Li and Yang, 2012; Seger and Peterson, 2013; Seger et al., 2015; Morriss et al., 2019). Converging findings indicate that the medial and dorsal prefrontal cortices, anterior cingulate cortex (ACC), posterior parietal cortex, and anterior insula play core roles in tracking categorization ambiguity and guiding adaptive behavior (Critchley et al., 2001; Paulus et al., 2001; Grinband et al., 2006; Grupe and Nitschke, 2013; Seger and Peterson, 2013; Seger et al., 2015; Paul et al., 2015).

For example, Grinband et al. (2006) reported that a network including the medial frontal gyrus, anterior insula, thalamus, and ventral striatum exhibited increasing activation as category uncertainty increased. This view is further supported by Paul et al. (2015), who used a one-dimensional perceptual-categorization task with an uncertainty-response option and found that declining to categorize—rather than task difficulty—activated a distributed network including the prefrontal cortex (PFC), anterior and posterior cingulate cortex (ACC, PCC), anterior insula, and posterior parietal cortex. This pattern indicates that uncertainty in categorization can trigger a metacognitive control process distinct from general associative difficulty. Davis et al. (2017) further demonstrated that feature-based category learning under uncertainty recruited lateral and inferior PFC, dorsomedial PFC, striatum, superior parietal cortex, and occipital regions, suggesting a distributed network supporting integration of probabilistic information for adaptive decisions. Morriss et al. (2019) conducted a meta-analysis across uncertainty tasks and found consistent bilateral anterior insula activation, highlighting its role in monitoring uncertainty. Feng et al. (2022) distinguished between risk, ambiguity, and threat anticipation and conducted meta-analyses to identify shared uncertainty-related regions. They found that although the right insula emerged as a common node across uncertainty states, ambiguity also recruited extensive frontal–parietal regions, and threat anticipation involved bilateral insula activity. Their findings indicate that uncertainty engages a distributed network rather than a strictly lateralized system, supporting the involvement of fronto-parietal and insular regions in uncertain decision making.

Together, these studies suggest that frontal–parietal regions and the insula form a core network for monitoring categorization uncertainty, with lateralization patterns depending on the task’s contextual and hierarchical structure. However, few studies have examined graded or hierarchical levels of uncertainty in category induction paradigms.

Importantly, uncertainty during categorization is closely related to—but not limited to—explicit confidence judgments. A growing body of work demonstrates that confidence- and uncertainty-related signals engage medial frontal, cingulate, and parietal regions and are supported by distributed control networks, even when confidence is not explicitly reported (Zizlsperger et al., 2014; Drugowitsch et al., 2019; Cohanpour et al., 2024; Asadpour and Wong-Lin, 2025). These findings suggest that uncertainty can be implicitly monitored during decision-making. Nevertheless, most existing studies have focused on explicit confidence ratings, leaving it unclear how uncertainty is represented during similarity-based category induction.

In the present study, we investigated the neural mechanisms underlying graded levels of categorization uncertainty using a feature-based category induction task that engages similarity-based categorization processes. Participants categorized novel visual stimuli based on perceptual feature similarity across multiple dimensions, with minimal reliance on semantic knowledge and limited working-memory demands. Consistent with similarity-based accounts of categorization (Davis et al., 2017), this task required judgments based on aggregated feature similarity, providing a framework for examining the contribution of fronto-parietal and insular networks to uncertainty processing during category induction.

We designed five levels of categorization uncertainty (C0–C4), with high uncertainty (C2) reflecting the greatest similarity-based conflict between the probe stimulus and the reference pair. This design contrasts with prior dichotomous (easy vs. difficult) paradigms, allowing a more systematic and comprehensive investigation of neural activity under graded uncertainty. Behavioral and neural evidence indicate that, when semantic processing fails, participants rely on readily discriminable features, comparing the number of shared features between the probe and reference pair to make category judgments (Goel and Dolan, 2000).

Accordingly, we anticipated activation in the fronto-parietal network—including the medial and middle frontal gyri (BA11, BA32, BA46) and inferior parietal lobule (BA40)—as well as in the insula (BA13), with activity scaling with categorization uncertainty (Grinband et al., 2006; Li and Yang, 2012; Davis et al., 2017; Morriss et al., 2019; Feng et al., 2022). In addition to regional activation, we employed psychophysiological interaction (PPI) analyses to examine task-dependent functional coupling within the fronto-parietal and salience networks, aiming to reveal how distributed regions interact to support attentional allocation, similarity-based feature integration, and adaptive category judgments under high uncertainty (Vincent et al., 2008; Farrar et al., 2018; Valdebenito-Oyarzo et al., 2024; Timashkov et al., 2025). This approach allows us to investigate both the localized and network-level neural mechanisms underlying graded uncertainty in similarity-based category induction, providing a framework for understanding how the brain dynamically adjusts cognitive control and attentional resources in response to the degree of categorization ambiguity.

## 2 Experimental procedures

### 2.1 Participants

Twenty healthy, right-handed college students (10 males and 10 females; mean age: 23 years; age range: 19–23 years) participated in the study. All participants were eligible for MRI scanning, having no metallic implants, no claustrophobia, and a head size compatible with the custom head coil. Furthermore, participants had no history of neurological or psychiatric disorders and were not using psychoactive medications. Data from one participant was excluded from analysis due to inadequate performance on the experimental tasks. Prior to the scanning session, informed consent was obtained from each participant, and the study received approval from the Ethical Review Board of the Psychology Department at Southwest University.

### 2.2 Materials and tasks

**Figure 1:**
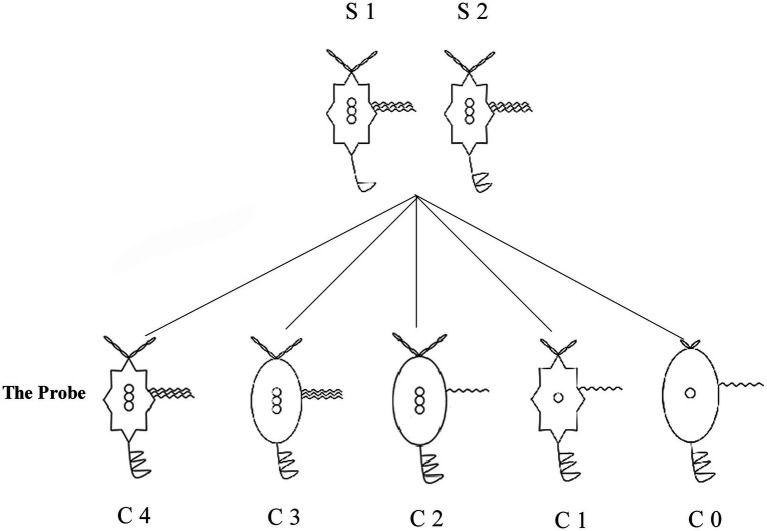
Experimental materials for each category induction condition. C0–C4 correspond to conditions 0–4, respectively. S1 and S2 denote stimulus 1 and stimulus 2. Stimuli varied across conditions to induce graded levels of similarity.

Novel animal stimuli were designed by the experimenter (Figure 1). The figures exhibited variations across five perceptual dimensions, each characterized by three attributes: body shape (round, octagonal, and 32-pointed star), head beard length (short, medium, and long), the number of tail amplitude (one, two, and three), the number of palm wave lines (one, two, and three), and the number of button (one, two, and three). (1) Category induction phase: three figures were presented simultaneously. The above pair of figures were members of the same category. Stimuli features varied randomly across five dimensions. Participants should extract the same features from the two figures. Participants were asked to judge whether the probe stimulus presented below belonged to the same category as the reference stimulus pair presented above. However, participants were not required to respond immediately in this phase. (2) Response phase: participants indicated their decision by pressing one of four buttons, arranged from left to right as follows: (a) yes (the same category), certain; (b) yes (the same category), slightly uncertain; (c) no (not the same category), slightly uncertain; (d) no (not the same category), certain. (3) Feedback phase: feedback was provided to indicate a correct or incorrect response depending on the participants’ choice.

The experiment comprised five conditions defined by feature overlap ratios: 4:0, 4:1, 4:2, 4:3, and 4:4, referred to as Condition 0 (C0) through Condition 4 (C4), respectively. Experimental conditions were defined by the number of shared perceptual features among the three stimuli. In all conditions, the two reference stimuli presented at the top of the display shared four common features. The conditions differed in the number of features shared between the probe stimulus and the reference pair. Specifically, in the 4:0 condition (C0), the probe stimulus shared no features with the reference pair. In the 4:1 (C1), 4:2 (C2), 4:3 (C3), and 4:4 (C4) conditions, the probe stimulus shared one, two, three, or four features with the reference pair, respectively. Accordingly, the probability that the probe stimulus belonged to the same category as the reference stimuli was set to 0, 0.25, 0.50, 0.75, and 1 for C0 through C4, respectively.

The feedback ratio was set according to the probability. Participants were not explicitly informed about the probabilities of receiving feedback. Instead, participants were informed that the probability of the probe stimulus belonging to the same category increased with its similarity to the reference stimulus pair.

### 2.3 Procedure

**Figure 2:**
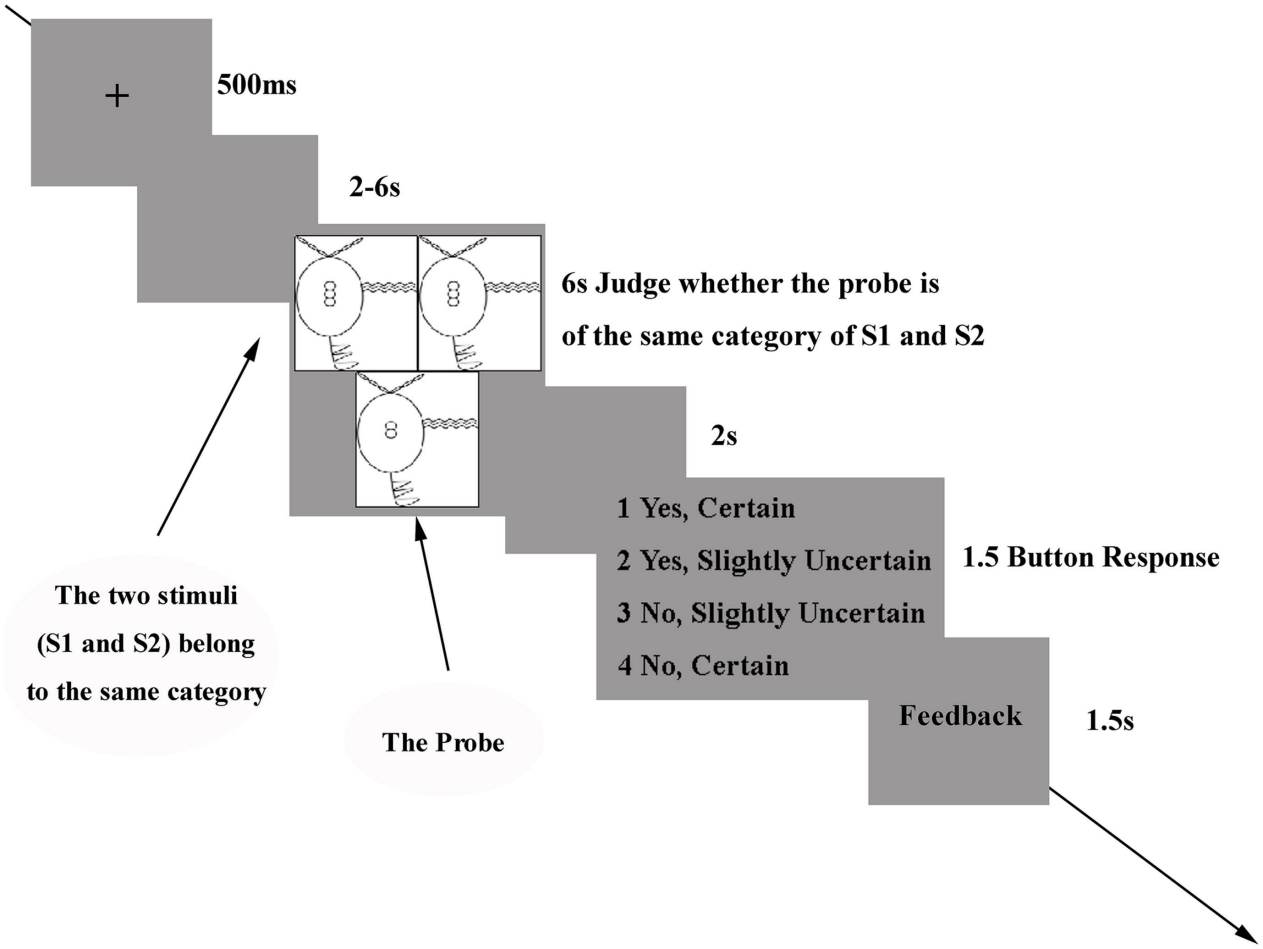
Experimental procedure. A fixation cross (500 ms) was first presented to stabilize gaze, followed by a blank screen lasting 2–6 s. Three stimuli (S1, S2, and the probe) were then presented simultaneously for 6 s to assess whether the probe belonged to the same category as S1 and S2, without requiring a motor response. After a 2 s blank screen, four response options were displayed for up to 1.5 s (from left to right: 1 = yes, certain; 2 = yes, slightly uncertain; 3 = no, slightly uncertain; 4 = no, certain) to indicate category membership and confidence. Each trial concluded with 1.5 s of accuracy feedback.

At the start of each trial, a fixation cross was displayed on the screen for 500 ms (Figure 2). Following a blank screen lasting 2 to 6 s, three stimuli (S1, S2, and probe) were presented simultaneously for 6 s. Participants were instructed not to provide any motor response and instead, to keep the attributes in mind for the subsequent categorization test. After a blank screen (2 s), four choices were displayed for up to 1.5 s, or until the participant responded. Participants were asked to judge whether the probe stimulus belonged to the same category as the reference stimuli and to indicate their level of certainty. Participants indicated their decision by pressing one of four buttons, arranged from left to right: (1) yes, certain; (2) yes, slightly uncertain; (3) no, slightly uncertain; (4) no, certain. Lastly, participants received feedback on their categorization for 1.5 s.

Participants completed four runs, each consisting of 45 trials. C0 and C4 each comprised 30 trials. Meanwhile, C1, C2, and C3 each contained 40 trials, yielding a total of 180 trials across the tasks. Each run encompassed all five conditions. Trials within each run were presented in a randomized order.

### 2.4 Behavioral data analysis

Behavioral measures were analyzed using a within-subjects one-way ANOVA with five measurement conditions. To assess the reliability of the sample size (*N* = 20), a power analysis was conducted using G*Power. The input parameters were: *α* = 0.05, desired power = 0.8, single group, five repeated measurements, correlation among repeated measures = 0.5, and nonsphericity correction *ε* = 1. The output results were: non-centrality parameter *λ* = 12.70, critical *F* = 2.492, numerator df = 4, denominator df = 76, and effect size *f* = 0.252. These results indicate that, despite the relatively small sample size, the within-subjects design provides reasonable power to detect medium effects.

To control for baseline differences in confidence reporting across participants, metacognitive sensitivity was quantified using signal detection theory-based measures. Objective stimulus discrimination sensitivity (*d*′), metacognitive sensitivity (meta-*d*′), and metacognitive efficiency (meta-*d*′/*d*′) were computed following established procedures (Maniscalco and Lau, 2012). These measures were used to characterize individual differences in confidence calibration and to control for baseline variability in subsequent brain–behavior analyses.

### 2.5 fMRI data acquisition

Functional magnetic resonance imaging (fMRI) data were acquired using a Siemens TRIO 3.0 T full-body MRI scanner (Siemens, Erlangen, Germany). Anatomical images (256 × 256 × 176) with 1 mm × 1 mm × 1 mm resolution were acquired for each participant using a T1-weighted three-dimensional magnetization prepared rapid gradient echo (MPRAGE) sequence, with parameters including an inversion time of 900 ms, a repetition time of 1,900 ms, an echo time of 2.52 ms, and a flip angle of 9 °. For functional scanning, an echo planar imaging (EPI) flip angle (90 °; field of view, 220 mm; in-plane resolution, 64 × 64; 0.99 mm gap; voxel size, 3.44 mm × 3.44 mm × 3 mm) with prospective acquisition correction (PACE) was used, which helped to minimize its impact on the data acquisition. Thirty-two axial slices were used to cover the entire cerebral cortex without gaps, and slices were positioned along the anterior commissure–posterior commissure plane.

### 2.6 fMRI data analyses

The data were analyzed using SPM12 (Wellcome Department of Imaging Neuroscience; https://www.fil.ion.ucl.ac.uk/spm/) to pre-process the functional images (Friston et al., 1997). Slice timing was applied to adjust slice order, followed by realignment to estimate and modify the six parameters of head movement. The first three images were discarded to achieve magnet-steady images. The images were then normalized to MNI space, with a voxel size of 3 × 3 × 3 mm^3^. Spatial smoothing was applied to the normalized data using a Gaussian kernel. The full width at half maximum (FWHM) was specified as 8 × 8 × 8 mm^3^.

Following pre-processing, we modeled 20 regressors per run (corresponding to fixation cross, category induction, response, and feedback phases across five conditions) to construct the design matrix. For each participant, we combined all four runs into a single general linear model (GLM). These regressors were convolved with the canonical hemodynamic response function, with six motion parameters from each participant included as linear confound regressors only; no temporal derivatives, quadratic expansions, or censoring/scrubbing procedures were applied. We generated 20 contrast images per participant by pooling data from the four runs.

These five contrasts for category induction were submitted to a one-way repeated-measures ANOVA to assess group-level random effects. The statistical threshold for the fMRI analyses was set at a familywise error rate (FWE) of *p* < 0.05, with a minimum cluster size of 20 contiguous voxels.

We investigated brain activity in specific regions of interest (ROIs), including the left medial prefrontal cortex (BA11), left cingulate gyrus (BA32), left middle frontal gyrus (BA46), left inferior parietal lobule (IPL, BA40), and left insula (BA13) during category induction to assess categorization uncertainty. Four ROIs were identified based on whole-brain FWE-corrected activations, while the IPL was defined *a priori* based on previous literature (Davis et al., 2017; Feng et al., 2022). ROIs were defined as spheres with a 6 mm radius centered on the specified coordinates. Mean beta values for each ROI corresponding to different event types were extracted from the first-level analysis of individual participants.

Beta values from all ROIs were submitted to one-way repeated-measures ANOVAs to assess statistical significance. For the four data-driven ROIs, pairwise comparisons between conditions were Bonferroni-corrected, and *p*-values <0.05 were considered significant. For the IPL, pairwise comparisons across the five conditions were Bonferroni-corrected (10 pairwise comparisons), and corrected *p* < 0.005 were considered significant.

Brain–behavior relationships were examined using correlation analyses at both the within-task and between-subject levels. First, Pearson correlations were conducted between reaction time and mean beta values extracted from each ROI, with Bonferroni correction applied to control for multiple comparisons. Second, to examine between-subject variability in uncertainty monitoring, correlations were performed between individual differences in metacognitive efficiency (meta-*d*′/*d*′) and mean beta values extracted from each ROI. Partial correlations controlling for objective stimulus discrimination sensitivity (*d*′) were additionally computed to assess associations independent of first-order task performance.

Although metacognitive accuracy (meta-*d*′) was computed at the behavioral level, metacognitive efficiency (meta-*d*′/*d*′) was selected for brain-behavior analyses because it provides a scale-invariant measure of metacognitive sensitivity while controlling for individual differences in perceptual discrimination ability (Maniscalco and Lau, 2012; Fleming, 2017). This approach allowed us to examine neural correlates of individual differences in uncertainty monitoring beyond baseline performance effects.

### 2.7 Psychophysiological interaction analysis

Based on the ROI analysis, five seed regions were selected for the PPI analysis: the left cingulate gyrus (MNI: −6, 24, 42), the left middle frontal gyrus (MNI: −42, 24, 24), the left medial frontal gyrus (MNI: −3, 33, −12), the left insula (MNI: −36, 6, 12), and the left inferior parietal lobule (IPL; MNI: −30, −42, 42). For the five seed regions, 6 mm-radius spheres were centered on the peak voxels identified in each participant’s activation maps. PPI regressors were constructed for the contrast of the most uncertain condition (C2) versus the average of the two most certain conditions ((C0 + C4)/2). The first-level GLM included the interaction term (PPI), the main effect of the seed region, the psychological regressors, and six motion parameters as regressors of no interest. Contrast images were entered into second-level one-sample *t*-tests.

To examine the relationship between task-modulated connectivity and behavior, regions showing significant connectivity at a voxel-wise threshold of *p* < 0.001, uncorrected, were further analyzed. For instance, a 6 mm-radius spherical ROI was defined at the peak of the left IPL cluster [MNI (−39, −66, 42)] that showed significant task-modulated connectivity with the left cingulate gyrus seed, using MarsBaR. Mean PPI *β*-values were then extracted from this ROI for each participant and correlated with behavioral reaction time to assess whether functional connectivity was associated with performance under uncertainty.

## 3 Results

### 3.1 Behavioral results

As shown in Table 1, participants’ categorization key responses were converted into confidence-weighted probability scores, representing the subjective probability that the probe stimulus belonged to the same category. The scores were assigned as follows: “yes, certain” = 1.00; “yes, slightly uncertain” = 0.75; “no, slightly uncertain” = 0.25; and “no, certain” = 0.00. Descriptive statistics showed that the mean probability systematically increased across conditions, from C0 (M = 0.04, SD = 0.04) to C4 (M = 0.97, SD = 0.06).

**Table 1:** Descriptive statistics of confidence-weighted probability scores across the five conditions.

Condition	M	SD
C0	0.04	0.04
C1	0.15	0.09
C2	0.42	0.15
C3	0.77	0.13
C4	0.97	0.06

M, mean; SD, standard deviation.

A one-way repeated-measures ANOVA revealed a significant main effect of condition, *F* (4, 72) = 331.42, *p* < 0.001, partial *η*^2^ = 0.95. Pairwise comparisons with Bonferroni correction indicated significant differences among all five conditions (all *p*-values < 0.001). These results demonstrate that participants’ category judgments varied systematically across the five conditions, consistent with the paired-comparison probability model.

As shown in Table 2, a one-way repeated-measures ANOVA revealed a significant main effect of condition on accuracy, *F* (4, 72) = 244.55, *p* < 0.001, partial *η*^2^ = 0.93. Pairwise comparisons with Bonferroni correction indicated that the accuracy rate in condition C2 was significantly lower than that in all other conditions (all *p*-values < 0.05). There were no significant differences in accuracy between C0 and C4, or between C1 and C3 (all *p*-values > 0.05). The accuracy rates in C0 and C4 were significantly higher than those in C1 and C3 (all *p*-values < 0.05).

**Table 2:** Accuracy rates and reaction times across experimental conditions.

Condition	Accuracy rate (M ± SD)	Reaction time (ms, M ± SD)
C0	0.98 ± 0.03	394.96 ± 62.64
C1	0.72 ± 0.05	420.10 ± 73.64
C2	0.50 ± 0.07	450.44 ± 103.04
C3	0.68 ± 0.09	424.25 ± 101.25
C4	0.99 ± 0.02	392.93 ± 71.92

A one-way repeated-measures ANOVA on reaction times revealed a significant main effect of condition, *F* (4, 72) = 6.00, *p* < 0.001, partial *η*^2^ = 0.25. Pairwise comparisons with Bonferroni correction indicated that the reaction time in C2 was significantly longer than that in C0, C3, and C4 (all *p*-values < 0.05). The differences between C0 and C1, and between C1 and C4, were also significant (all *p*-values < 0.05). Overall, these results indicate that the greater the uncertainty of categorization, the lower the accuracy and the longer the reaction time.

To directly assess potential asymmetry between C1 and C3, we first conducted one-sample *t*-tests comparing accuracy in each condition against chance level (50%). Both C1 [*t* (37) = 32.45, *p* < 0.001] and C3 [*t* (37) = 32.09, *p* < 0.001] were significantly above chance. Importantly, to test whether the magnitude of deviation from chance differed between the two conditions, we conducted a paired-samples *t*-test on the values of (Accuracy − 50%). This comparison did not reach statistical significance [*t* (18) = 1.66, *p* = 0.11], indicating no reliable asymmetry in overall accuracy between these theoretically symmetric levels of feature overlap.

To further examine whether accuracy patterns were influenced by response strategy, signal detection theory analyses were conducted. Discrimination sensitivity (*d*′) did not differ significantly between C1 (M = 0.24, SD = 0.55) and C3 [M = −0.36, SD = 0.55; *t* (36) = 1.54, *p* = 0.13], indicating comparable objective task difficulty. In contrast, response criterion (c) differed robustly between conditions, with a conservative bias in C1 (M = 1.29, SD = 0.47) and a liberal bias in C3 [M = −1.25, SD = 0.66; *t* (36) = 13.77, *p* < 0.001]. These findings suggest that participants adjusted their decision thresholds across similarity levels; however, these criterion shifts did not translate into a statistically reliable asymmetry in accuracy.

To further account for potential baseline differences in confidence reporting across participants, we computed signal detection theory indices of objective stimulus discrimination sensitivity (*d*′) and metacognitive efficiency (meta-*d*′/*d*′) (see Table 3). Participants showed above-chance objective discrimination performance [*d*′: M = 2.01, SD = 0.81; *t* (16) = 10.72, *p* < 0.001], and metacognitive accuracy was also significantly greater than zero [meta-*d*′: M = 0.65, SD = 0.69; *t* (16) = 4.37, *p* < 0.001]. Mean metacognitive efficiency (meta-*d*′/*d*′) was 0.28 (SD = 0.26), within the typical range reported in prior metacognition studies. These measures were included to control for individual differences in confidence reporting rather than as primary variables of interest in subsequent neuroimaging analyses.

**Table 3:** Objective stimulus discrimination sensitivity (*d*′), metacognitive accuracy (meta-*d*′), and metacognitive efficiency (meta-*d*′/*d*′) across participants.

Measure	M	SD	*t*	*p*	Range
Objective stimulus discrimination sensitivity (*d*′)	2.01	0.81	10.72	0.001	0.15–3.28
Metacognitive accuracy (meta-*d*′)	0.65	0.69	4.37	0.001	0.00–2.18
Metacognitive efficiency (meta-*d*′/*d*′)	0.28	0.26	4.72	0.001	0.00–0.78

All *t*-tests examined whether the measure was significantly greater than zero. Data were based on 17 valid participants (excluding two participants due to missing error trials).

### 3.2 Imaging results

**Figure 3:**
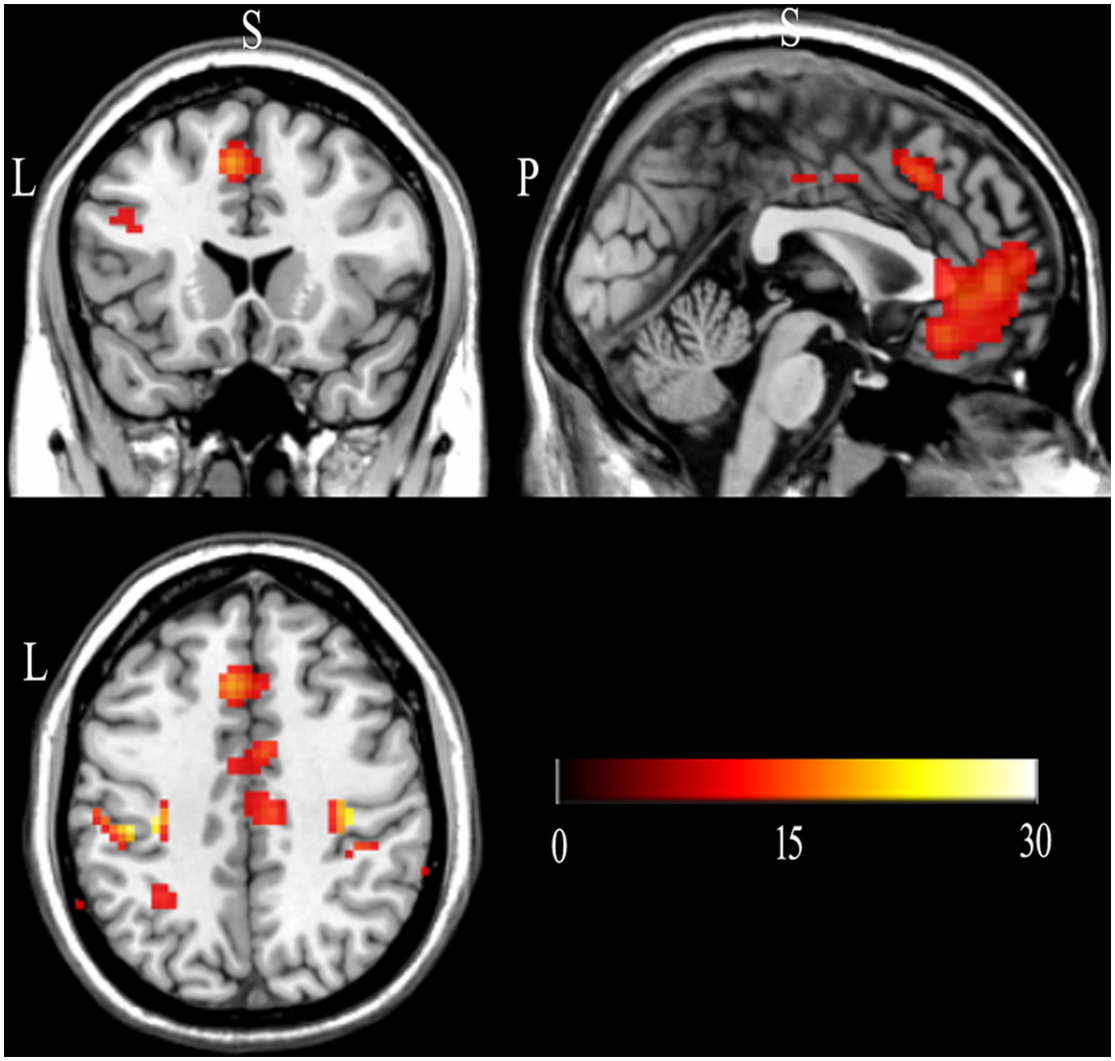
Brain activity during category induction phase: a one-way within-subject ANOVA (FWE-corrected, *p* < 0.05). L, left; S, superior; P, posterior.

As shown in Table 4 and Figure 3, the one-way within-subject ANOVA revealed extensive activation in the left precentral gyrus (BA4), right postcentral gyrus (BA2), left medial frontal gyrus (BA11), left cingulate gyrus (BA32), left middle frontal gyrus (BA46), bilateral dentate, and left insula (BA13).

**Table 4:** Whole-brain results from the one-way within-subject ANOVA.

Area	BA	*x*	*y*	*z*	*F*	Voxel of cluster (*k*)
Left precentral gyrus	4	−39	−21	57	42.99	708
Right postcentral gyrus	2	48	−21	45	29.47	1362
Left medial frontal gyrus	11	−3	33	−12	15.77	565
Left medial frontal gyrus	10	−6	54	9	15.61	
Left cingulate gyrus	32	−6	24	42	18.17	134
Left superior frontal gyrus	8	−6	18	48	18.07	
Left middle frontal gyrus	46	−42	24	24	15.98	88
Left middle frontal gyrus	9	−42	12	27	11.81	
Left anterior lobe, dentate		−18	−54	−21	22.45	121
Right anterior lobe, dentate		18	−51	−21	34.55	129
Left sub-lobar, insula	13	−36	6	12	14.27	42
Left sub-lobar, insula	13	−39	−18	18	15.36	29

FWE *p* < 0.05. L, left.

**Figure 4:**
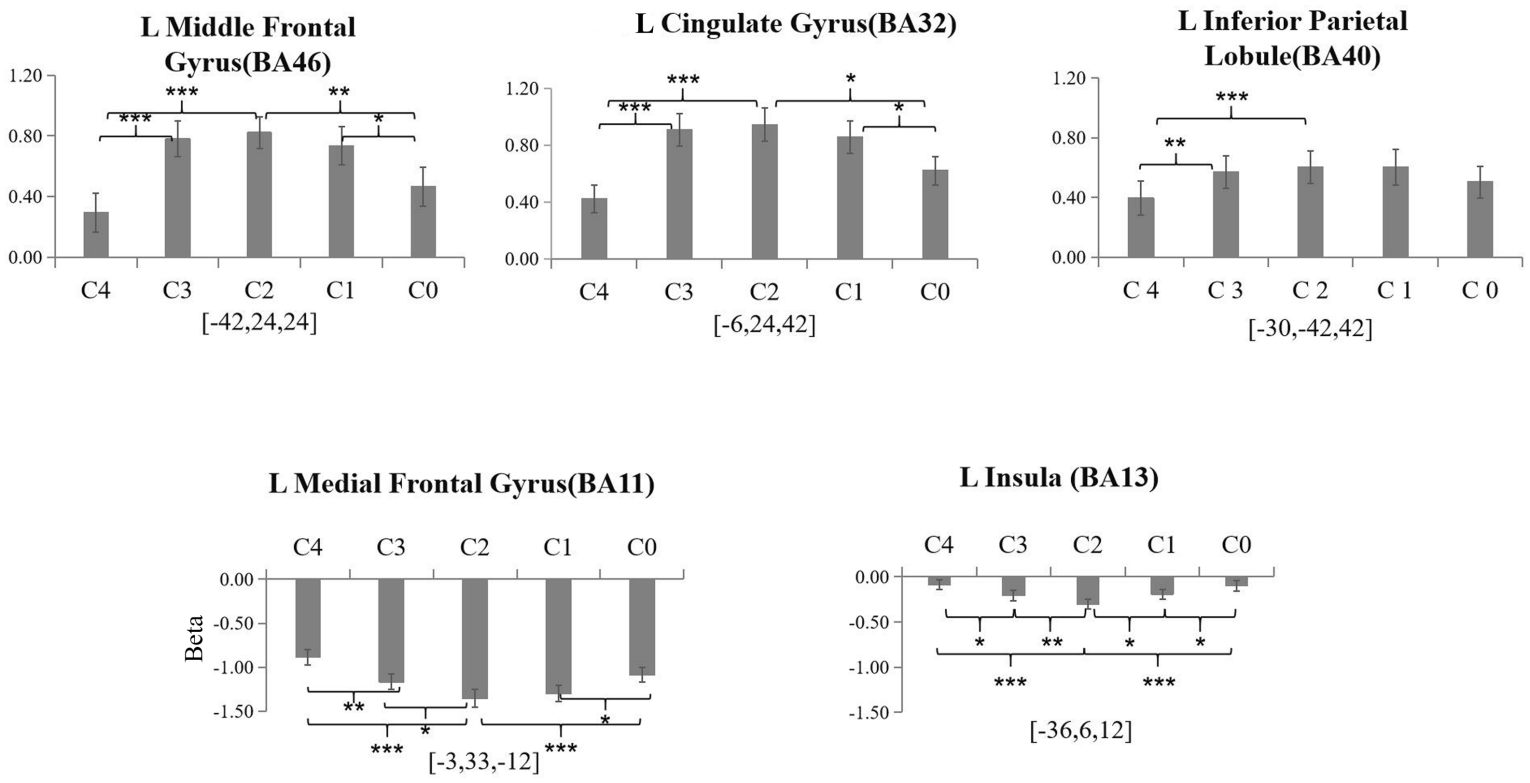
Beta means within five ROIs for category induction among five conditions (C4, C3, C2, C1, C0). Error bars represent SE of the mean across all participants. L, left. ^*^p < 0.05, ^**^p < 0.01, and ^***^p < 0.001.

Based on the brain activation results of the present study and previous findings, we analyzed five regions of interest (ROIs; Figure 4): the left medial frontal gyrus (BA11), left cingulate gyrus (BA32), left middle frontal gyrus (BA46), left inferior parietal lobule (BA40), and left insula (BA13).

For the left cingulate gyrus (BA32), a one-way within-subject ANOVA also revealed a significant main effect of condition, *F* (4, 72) = 21.35, *p* < 0.001, partial *η*^2^ = 0.54. *Post-hoc* pairwise comparisons showed that positive activation in C2 was significantly greater than in C4 and C0 (all *p*-values < 0.05). Activation in C3 was significantly greater than in C4 (*p* < 0.001). Activation in C1 was significantly greater than in C0 (*p* < 0.05).

For the left middle frontal gyrus (BA46), a one-way within-subject ANOVA revealed a significant main effect of condition, *F* (4, 72) = 18.40, *p* < 0.001, partial *η*^2^ = 0.51. *Post-hoc* pairwise comparisons showed that beta activation in C2 was significantly greater than in C4 and C0 (all *p*-values < 0.01). Activation in C3 was significantly greater than in C4 (*p* < 0.001), activation in C1 was significantly greater than in C0 (*p* < 0.05).

For the left inferior parietal lobule (BA40), a one-way within-subject ANOVA showed a significant main effect of condition, *F* (4, 72) = 9.58, *p* < 0.001, partial *η*^2^ = 0.35. *Post-hoc* pairwise comparisons indicated that activation in C2 was significantly greater than in C4 (*p* < 0.001). Activation in C3 was significantly greater than in C4 (*p* < 0.005).

For the left medial frontal gyrus, a one-way within-subject ANOVA showed a significant main effect of condition, *F* (4, 72) = 18.22, *p* < 0.001, partial *η*^2^ = 0.50. *Post-hoc* pairwise comparisons indicated that negative activation in C2 was significantly greater than in C4, C3, and C0 (all *p*-values < 0.05). Negative activation in C3 was significantly greater than in C4 (*p* < 0.01), and activation in C1 was significantly greater than in C0 (*p* < 0.05).

For the left insula (BA13), a one-way within-subject ANOVA showed a significant main effect of condition, *F* (4, 72) = 13.60, *p* < 0.001, partial *η*^2^ = 0.43. *Post-hoc* pairwise comparisons revealed that negative activation in C2 was significantly greater than in the other four conditions (all *p*-values < 0.05). Negative activation in C3 was significantly greater than in C4 (*p* < 0.05), and negative activation in C1 was significantly greater than in C0 (*p* < 0.05).

Correlation analyses revealed that only the left insula showed a significant association with reaction time after Bonferroni correction (*r* = −0.49, *p* < 0.01). No other regions showed significant associations after correction.

To examine between-subject variability, we conducted an exploratory analysis testing whether individual differences in metacognitive efficiency were associated with task-related neural activation. Partial correlations (controlling for objective discrimination ability, *d*′) were computed between metacognitive efficiency (meta-*d*′/*d*′) and mean beta values extracted from the five ROIs.

This analysis revealed a marginal negative association between metacognitive efficiency and activation in the left middle frontal gyrus (BA46) (*r* = −0.47, *p* = 0.051, one-tailed), such that higher metacognitive efficiency was associated with lower activation in this region. No significant correlations were observed in the remaining ROIs (all *p*-values > 0.05).

These results are reported as exploratory and are intended to address individual variability in confidence-related behavior rather than to establish a primary neural correlate of metacognitive ability.

### 3.3 PPI results

#### 3.3.1 Task-modulated functional connectivity of seed regions

**Figure 5:**
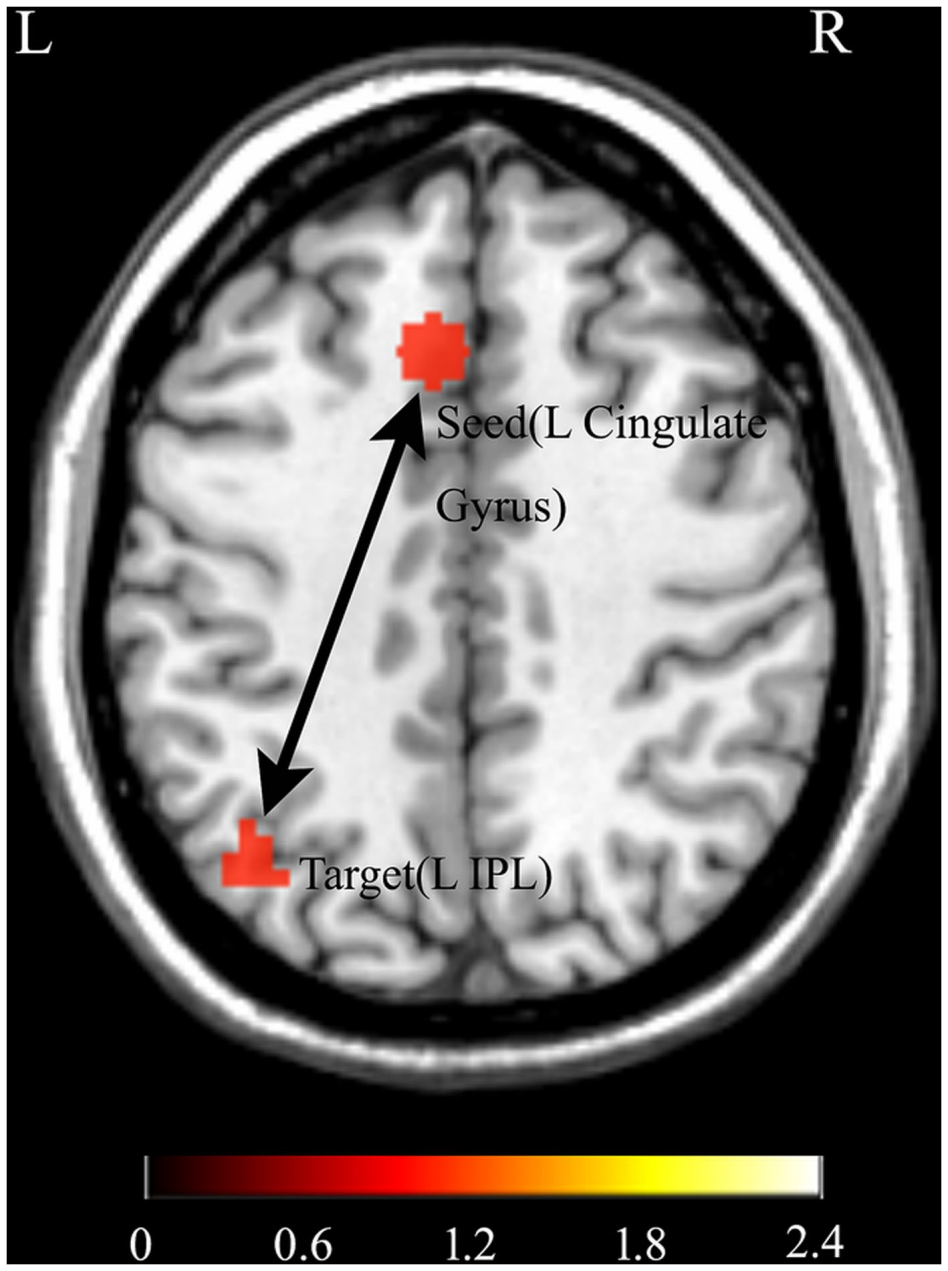
Task-modulated functional connectivity revealed by psychophysiological interaction (PPI) analysis. Increased coupling was observed between the left cingulate gyrus and the left inferior parietal lobule under high uncertainty (C2) compared with low uncertainty conditions (C0 and C4; voxel-wise threshold *p* < 0.001, uncorrected).

PPI analyses were conducted for five seed regions [left cingulate gyrus, left medial frontal gyrus, left middle frontal gyrus, left insula, and left inferior parietal lobule (IPL)] to examine changes in functional connectivity associated with uncertainty (C2 vs. C0 and C4 conditions). As shown in Figure 5, at a voxel-wise threshold of *p* < 0.001 (uncorrected), significant connectivity was observed only for the left cingulate gyrus (BA32) seed. Specifically, the left cingulate gyrus seed showed significant connectivity with the left inferior parietal lobule (BA39) [MNI: (−39, −66, 42); cluster size = 45; *T* = 5.07].

The other seed regions (left medial frontal gyrus, middle frontal gyrus, insula, and IPL) did not show significant connectivity at this threshold. Exploratory analyses using a more liberal threshold (*p* < 0.05) suggested weak connectivity patterns, but these were not considered statistically robust.

#### 3.3.2 ROI-based PPI *β*-values and behavioral correlations

A 6-mm-radius spherical ROI was defined at the peak of the left inferior parietal lobule (IPL) cluster showing significant connectivity with the left cingulate gyrus seed. Mean PPI *β*-values were extracted from this ROI for each participant and correlated with behavioral reaction time. The correlation was not significant (*r* = 0.24, *p* = 0.36), indicating that left cingulate gyrus-IPL connectivity under uncertainty was not directly associated with task performance.

## 4 Discussion

The behavioral results were highly consistent with the feature-based similarity structure of the task. Participants’ confidence-weighted probability scores increased systematically as the number of shared features between the probe and the category-defining pair increased from C0 through C4, reflecting a graded perception of category similarity. Performance in C2 was characterized by the lowest accuracy and the longest reaction times among the five conditions, suggesting that intermediate similarity levels evoked the greatest uncertainty in category comparisons, as the probe stimulus was neither clearly inside nor clearly outside the category-defined group. In contrast, performance in the extreme conditions (C0 and C4) showed maximal dissimilarity or maximal similarity, leading to fast, consistent responses.

Although C1 and C3 were theoretically symmetric in terms of feature overlap, additional analyses revealed no statistically reliable asymmetry in overall accuracy between the two conditions. Signal detection analyses indicated differences in response criterion, with a more conservative bias in C1 and a more liberal bias in C3; however, discrimination sensitivity did not differ significantly. These findings suggest minor adjustments in decision threshold under graded similarity, without altering the structural symmetry of task performance.

Importantly, the overall behavioral pattern remained graded, with maximal uncertainty at intermediate similarity levels. Therefore, these response characteristics do not affect the interpretation of the graded uncertainty effects observed in the neuroimaging data. Overall, participants’ judgments were guided by feature similarity, with greatest difficulty at intermediate levels.

### 4.1 Whole-brain activation modulated by perceptual categorization uncertainty

In the present study, a subset of regions of interest (ROIs)—including the left medial frontal gyrus (BA11), left cingulate gyrus (BA32), left middle frontal gyrus (BA46), left insula (BA13), and left inferior parietal lobule (BA40)—was selected based on whole-brain one-way repeated-measures ANOVA across the five categorization conditions (C0–C4). Beta values extracted from these ROIs systematically varied with categorization uncertainty. Notably, the left medial frontal gyrus (BA11) and left insula (BA13) showed negative activations, whereas the left cingulate gyrus (BA32), left middle frontal gyrus (BA46), and IPL (BA40) displayed positive activations. Other regions identified in whole-brain analyses, such as the left precentral gyrus (BA4), right postcentral gyrus (BA2), and bilateral dentate, did not show uncertainty-dependent modulation.

Negative activations in the left medial frontal gyrus (BA11) and left insula (BA13) likely reflect distinct functional mechanisms. The medial frontal gyrus, a core hub of the default mode network (DMN), may show task-induced deactivation to suppress internally oriented or self-referential processes, facilitating engagement of task-relevant networks (Raichle et al., 2001; McKiernan et al., 2003; Fan et al., 2014; Skowron et al., 2025). Similarly, the left insula, a key node of the salience network involved in uncertainty monitoring and interoception (Fan et al., 2014; Morriss et al., 2019; Feng et al., 2022), showed reduced activation, which was significantly correlated with participants’ reaction times. This suggests that, under high uncertainty in similarity-based categorization, insular deactivation may reflect a shift from internal monitoring to externally oriented attentional allocation, prioritizing similarity comparison and category judgment.

Most notably, positive activations were observed in the left cingulate gyrus (BA32), left middle frontal gyrus (BA46), and left IPL (BA40), with activity increasing alongside categorization uncertainty. The left cingulate gyrus (BA32), part of the dorsal anterior cingulate cortex (dACC), has been implicated in monitoring decision conflict, integrating cognitive and autonomic signals, and signaling the need for adaptive control under uncertainty (Critchley et al., 2001; Morriss et al., 2019). These regions collectively form key nodes of the fronto-parietal control network, supporting attentional allocation, working memory, and adaptive decision-making under conditions of perceptual categorization uncertainty (Grinband et al., 2006; Seger and Peterson, 2013; Seger et al., 2015; Paul et al., 2015; Davis et al., 2017; Feng et al., 2022). The middle frontal gyrus (BA46) contributes to maintaining task-relevant information and selecting appropriate responses, while the IPL integrates stimulus features and guides attention to the most relevant information for category judgment (McCarthy et al., 1997; Bunge et al., 2002; Huettel et al., 2005).

Across the five categorization conditions, these ROIs’ activation patterns closely tracked the gradient of uncertainty rather than simply reflecting general executive demand, as maximal engagement occurred in the high-uncertainty condition (C2) rather than in the most demanding inhibitory control conditions. Importantly, these ROI beta values serve primarily to illustrate the trend of uncertainty-related activation. This whole-brain pattern provides a macro-level context for our PPI results, in which the left cingulate cortex (BA32) exhibited increased coupling with the IPL under high uncertainty, highlighting coordinated network-level mechanisms for resolving similarity-based categorization uncertainty and supporting adaptive category judgment.

The present findings can be situated within the broader literature on perceptual uncertainty and confidence in decision making. Prior work has shown that uncertainty-related signals engage medial frontal and parietal regions and are supported by distributed control networks, even when confidence is not explicitly reported (Zizlsperger et al., 2014; Drugowitsch et al., 2019; Cohanpour et al., 2024; Asadpour and Wong-Lin, 2025). In the present study, participants did not provide explicit confidence ratings during the category induction phase, and the observed activations in medial frontal, cingulate, and insular regions are therefore more appropriately interpreted as neural markers of similarity-based categorization uncertainty and associated control demands, rather than metacognitive confidence per se. In this sense, fronto-parietal and salience-related networks may support implicit uncertainty monitoring during category induction.

An exploratory analysis revealed a marginal association between activation in the left middle frontal gyrus (BA46) and metacognitive efficiency. Although this effect should be interpreted cautiously, it is consistent with the proposed role of the lateral prefrontal cortex in higher-order monitoring processes and does not alter the primary interpretation of the task-related uncertainty effects.

### 4.2 Task-modulated functional connectivity (PPI)

Our findings demonstrate that under high uncertainty, the left cingulate cortex (BA32) shows increased functional coupling with the left inferior parietal lobule (IPL), consistent with the engagement of fronto-parietal pathways supporting the integration of cognitive control and attentional processes. This aligns with models positing that uncertainty engages a distributed network including ACC/medial PFC and IPL (Valdebenito-Oyarzo et al., 2024). A recent meta-analysis of fMRI studies on uncertainty processing highlighted IPL as one of the most consistently activated regions, underscoring its role in integrating information, cognitive control, and goal-directed attention, complementing our PPI findings (Timashkov et al., 2025). Moreover, evidence from intrinsic functional connectivity studies (Vincent et al., 2008) supports the notion that ACC, lateral PFC, and IPL form core nodes of a fronto-parietal control system, anatomically positioned to integrate information from attention- and memory-related networks, providing a structural and functional basis for the ACC–IPL coupling observed under uncertainty. Further support comes from network analyses of task-based fMRI (Farrar et al., 2018), which showed that node strength in the left IPL and right caudal ACC significantly increased under uncertain compared with certain conditions, indicating that these regions enhance their connectivity within a broader fronto-parietal network to support adaptive processing under uncertainty.

ACC has been suggested to integrate diverse sources of uncertain information in cognitive tasks, supporting adaptive decision-making under uncertainty (Alexander et al., 2023). In our task, the left cingulate cortex (BA32) may monitor the high similarity-based categorization uncertainty associated with the C2 condition and coordinate attentional allocation in the IPL to support similarity comparison and adaptive category judgment. This provides a mechanistic explanation for the observed ACC-IPL coupling, highlighting the fronto-parietal network’s role in resolving high similarity-based categorization uncertainty, particularly in contrast to the low-uncertainty conditions (C0 and C4).

No significant association was found between connectivity strength and reaction time, consistent with prior research showing that neural coupling often reflects latent cognitive processes rather than overt behavior. In sum, our results highlight the role of ACC-IPL connectivity in categorization under uncertainty, indicating that the fronto-parietal control network dynamically allocates attentional and cognitive resources to support adaptive decision-making.

### 4.3 Limitations

The present study included a relatively small sample (*N* = 20), which limits the generalizability of the findings. However, the power analysis using G*Power indicated that for medium effects (*f* = 0.252), the within-subjects design retains reasonable statistical power. Therefore, the current findings constitute preliminary exploratory evidence, and future studies with larger sample sizes are warranted to confirm these results.

## 5 Conclusion

In summary, categorization uncertainty selectively engaged a fronto-parietal network comprising the left cingulate cortex, middle frontal gyrus, and inferior parietal lobule, all of which showed increasing activation with rising uncertainty. In contrast, decreased activation in the left medial frontal gyrus and insula—regions linked to the default mode and salience networks—suggests a shift away from internally oriented and interoceptive processing toward externally focused similarity evaluation. Furthermore, enhanced functional coupling between the left cingulate cortex and IPL under high uncertainty indicates that resolving uncertain similarity-based category information relies on coordinated interactions within this control network. Together, these findings highlight a dynamic neural mechanism through which the brain integrates attentional and control processes to support adaptive decisions under categorization uncertainty.

## Data Availability

The original contributions presented in the study are included in the article/supplementary material, further inquiries can be directed to the corresponding authors.
